# Case Report: Trametinib in the treatment of patients with metastatic lung adenocarcinoma harboring *NF1* mutation: a case series and literature review

**DOI:** 10.3389/fonc.2025.1481284

**Published:** 2025-10-15

**Authors:** Floryane Kim, Maxime Borgeaud, Claudio De Vito, Petros Tsantoulis, Alfredo Addeo

**Affiliations:** ^1^ Oncology Service, University Hospital Geneva, Geneva, Switzerland; ^2^ Precision Oncology, University Hospital Geneva, Geneva, Switzerland; ^3^ Pathology Department, University Hospital Geneva, Geneva, Switzerland

**Keywords:** trametinib, NF1 mutation, non-small-cell lung cancer, targeted therapy, case report

## Abstract

**Background:**

The neurofibromin 1 (NF1) protein regulates the downstream RAS/RAF/MEK/ERK pathway and functions as a tumor suppressor. Somatic pathogenic mutations in *NF1* are found in approximately 4.7%–10% of NSCLC, with a higher frequency in lung adenocarcinomas, reaching up to 15% in certain cohorts. Trametinib, a MEK inhibitor, has demonstrated activity in tumors with *NF1* alteration in preclinical models, and clinical activity in low-grade glioma and plexiform neurofibromas in neurofibromatosis type 1. Trametinib had only limited clinical efficacy in other tumor types with *NF1* mutations in the NCI-Match trial. However, the sole NSCLC patient that was evaluable for response in the NCI-Match trial benefited from a deep partial response. More data for the activity of MEK inhibitors in *NF1* altered NSCLC are needed.

**Cases presentation:**

We report here a series of four NSCLC patients with *NF1* pathogenic mutations treated with trametinib. All patients underwent extensive molecular testing with next-generation sequencing (custom 462-gene panel) and copy number variation analysis and were deemed to have potential *NF1*-loss-driven tumors after a case discussion in a multidisciplinary molecular tumor board. Two patients exhibited homozygous *NF1* LOF alterations, whereas two patients had heterozygous loss-of-function alterations. All patients were treated with oral trametinib 2 mg once daily, after failure of standard therapies. Trametinib was administered for a maximum duration of 9 weeks. The best response observed was a stable disease in one patient. All patients died within 3 months of treatment initiation. No side effects warranted treatment cessation.

**Conclusion:**

In this small case series, NSCLC patients with *NF1* alterations did not derive clinical benefit from trametinib. While these data do not support trametinib as a treatment option for *NF1*-mutated NSCLC, larger studies are required to draw firm conclusions.

## Introduction

The neurofibromin 1 (*NF1*) gene encodes for a small GTPase-activating protein that binds to the RAS family of proteins and functions as a tumor-suppressor gene. NF1 binds to KRAS, HRAS, and NRAS favoring their inactive GDP-bound state, thus governing cellular growth and differentiation (1). Germline *NF1* mutation is associated with neurofibromatosis type 1, characterized by the development of neurofibromas. Individuals with this syndrome also face an increased risk of various cancers, including malignant peripheral nerve sheath tumor (MPNST), leukemia, glioma, and breast cancer (2). Acquired somatic mutations in the *NF1* gene have been identified in a diverse array of malignancies that lack any connection with neurofibromatosis type 1 (3). Notably approximately 8% of non-small cell lung cancers (NSCLC) carry pathogenic mutations in *NF1 (* 4 *)*, although most studies do not report the percentage of patients with complete inactivation of both alleles, which is probably lower.

The enhanced understanding of tumor biology and the identification of drivers in NSCLC have enabled the development of targeted therapies that have revolutionized the management of these patients and improved outcomes. Trametinib, a mitogen-activated protein kinase (MEK) inhibitor targeting MEK1 and MEK2, crucial components of the MAPK/ERK pathway, has emerged as a potential treatment of *NF1* altered cancers (5, 6). It showed promising results in patients with unresectable plexiform neurofibromas and low-grade glioma in neurofibromatosis type 1 (5). However, the efficacy of trametinib in other tumors harboring *NF1* mutations appears limited. In the NCI-MATCH trial, which included patients with *NF1*-, *GNAQ-*, or *GNA11-* mutant tumors, three patients with *NF1*-mutated NSCLC were initially enrolled, but only one was evaluable for response, showing a near-complete response. The other two patients were not evaluable due to rapid disease progression or early withdrawal (7). We are not aware of other studies or case reports detailing the response of *NF1*-altered non-small cell lung cancer to MEK inhibitors. It is therefore unclear whether a clinically meaningful benefit can be derived from MEK inhibitors in this setting.

We present a series of four patients with *NF1* altered metastatic lung adenocarcinoma treated with trametinib in our institution, and a comprehensive review of the literature.

## Case presentation

### Molecular analysis

**Figure 1:**
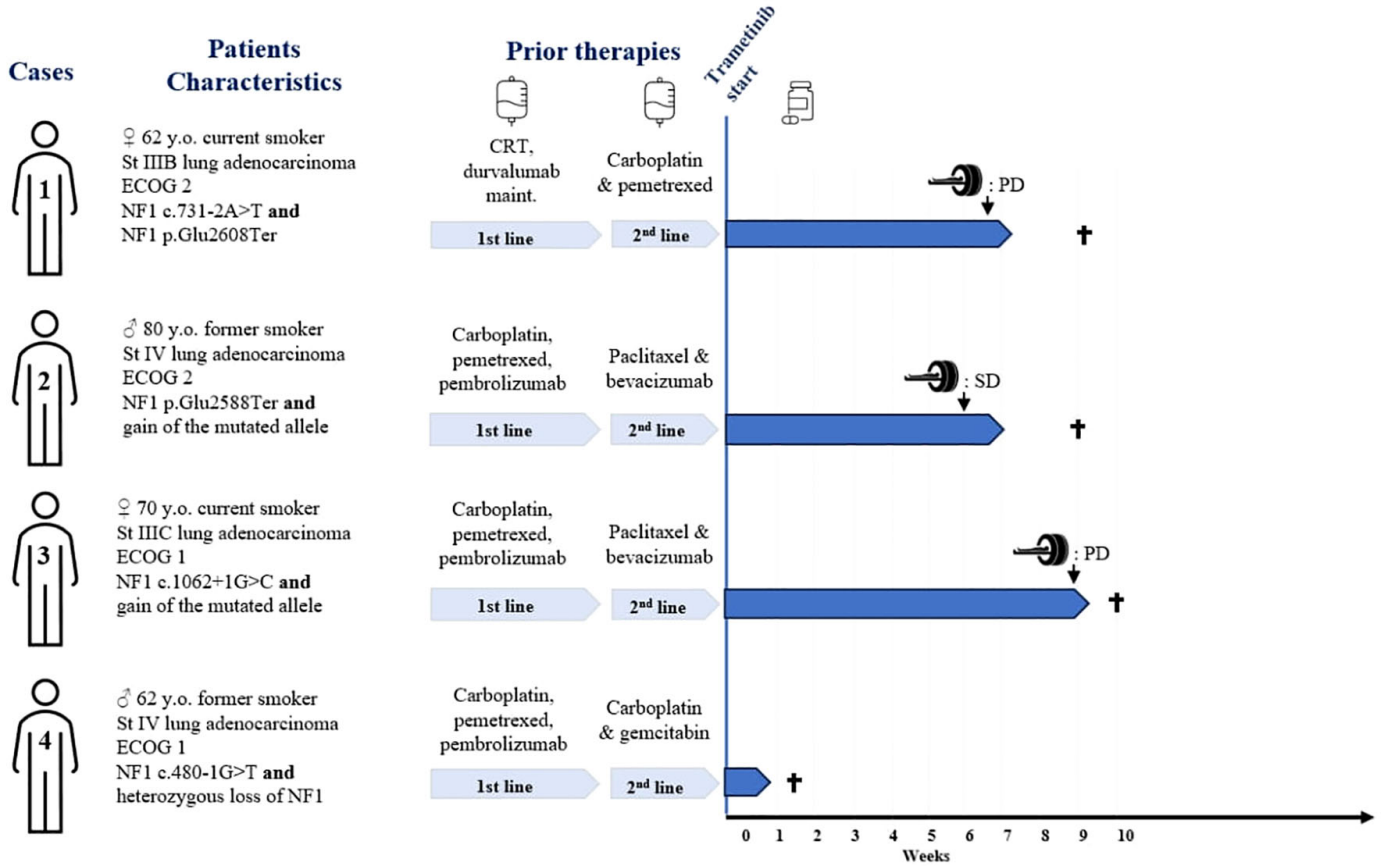
Patients' characteristics, and treatment outcomes. St, stage; y.o., years old; CRT, concurrent chemoradiotherapy; PD, progressive disease per RECIST criteria; SD, stable disease per RECIST criteria. † = death.

**Figure 2:**
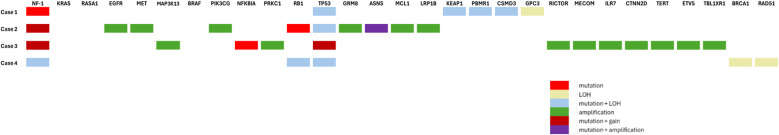
Genomic alterations in four patient cases.

Within our institution, patients with NSCLC progressing after first-line systemic therapy are systematically discussed at the molecular tumor board (TB). The four patients described below underwent the same extensive molecular analysis through next-generation sequencing (custom 462 gene panel, SureSelectHS capture-based Agilent) and copy number variations analysis (Oncoscan Assay kit, cat. 902,695; Thermo Fisher Scientific) as previously described (8). These analyses are routinely performed before discussion at our institution TB. The size of the NGS panel is >1 Mb. The Cancer Gene Census, COSMIC (Catalogue of Somatic Mutations In Cancer) (9), CIViC (Clinical Interpretations of Variants in Cancer) (10), and OncoKb (11) were used for variant interpretation and classification. For NF1 splice variant mutations, pathogenicity was determined based on SpliceAI (12) and Pangolin (13) algorithms. The clinical characteristics and treatment outcomes are summarized in **Figure 1**. A description of the different genomic alterations found for each patient is provided in **Table 1** and **Figure 2**.

**Table 1:** Molecular alterations in patients.

	SNV (NF1)	% of tumor	VAF	Mutation type	CNV	LOF vs. heterozygous NF1
Patient 1	c.731-2A>T	60%	24%	Splice acceptor variant	/	Biallelic LOF
	p.Glu2608Ter	/	28%	Stop codon	/	
Patient 2	p.Glu258Ter	50%	68%	Stop codon	Gain (four copies)	Monoallelic LOF
Patient 3	c.1062 + 1G>C	20%	21%	Splice donor variant	Gain (three copies)	Monoallelic LOF
Patient 4	c.480-1G>T	40%	28%	Splice acceptor variant	Heterozygous loss	Biallelic LOF

SNV, single-nucleotide variants; CNV, copy number variation. VAF, variant allele frequency; LOF, loss of function.

Trametinib was proposed for each case after discussion in the molecular tumor board based on preclinical rationale, suggesting that MEK inhibition can be effective in *NF1*-deficient tumors (14, 15), and by extrapolation from evidence in other *NF1*-altered cancers (6). The off-label use of trametinib and its therapeutic trial nature were clearly discussed with each patient individually. As this was an off-label use, approval for coverage was obtained from the health insurance on a case-by-case basis.

### Case 1

The first case was a 62-year-old woman and active smoker, diagnosed with stage IIIB according to the 8^th^ edition of the TNM, PD-L1 negative lung adenocarcinoma in September 2020. She underwent concomitant chemotherapy (carboplatin and paclitaxel) and radiotherapy, followed by maintenance durvalumab for 1 year. Two months after stopping immunotherapy, the patient presented with peritoneal metastatic relapse, biopsy-proven. First-line chemotherapy was started with carboplatin and pemetrexed allowing a stable disease, followed by pemetrexed maintenance, until a new metastatic progression in November 2022.

**Figure 3:**
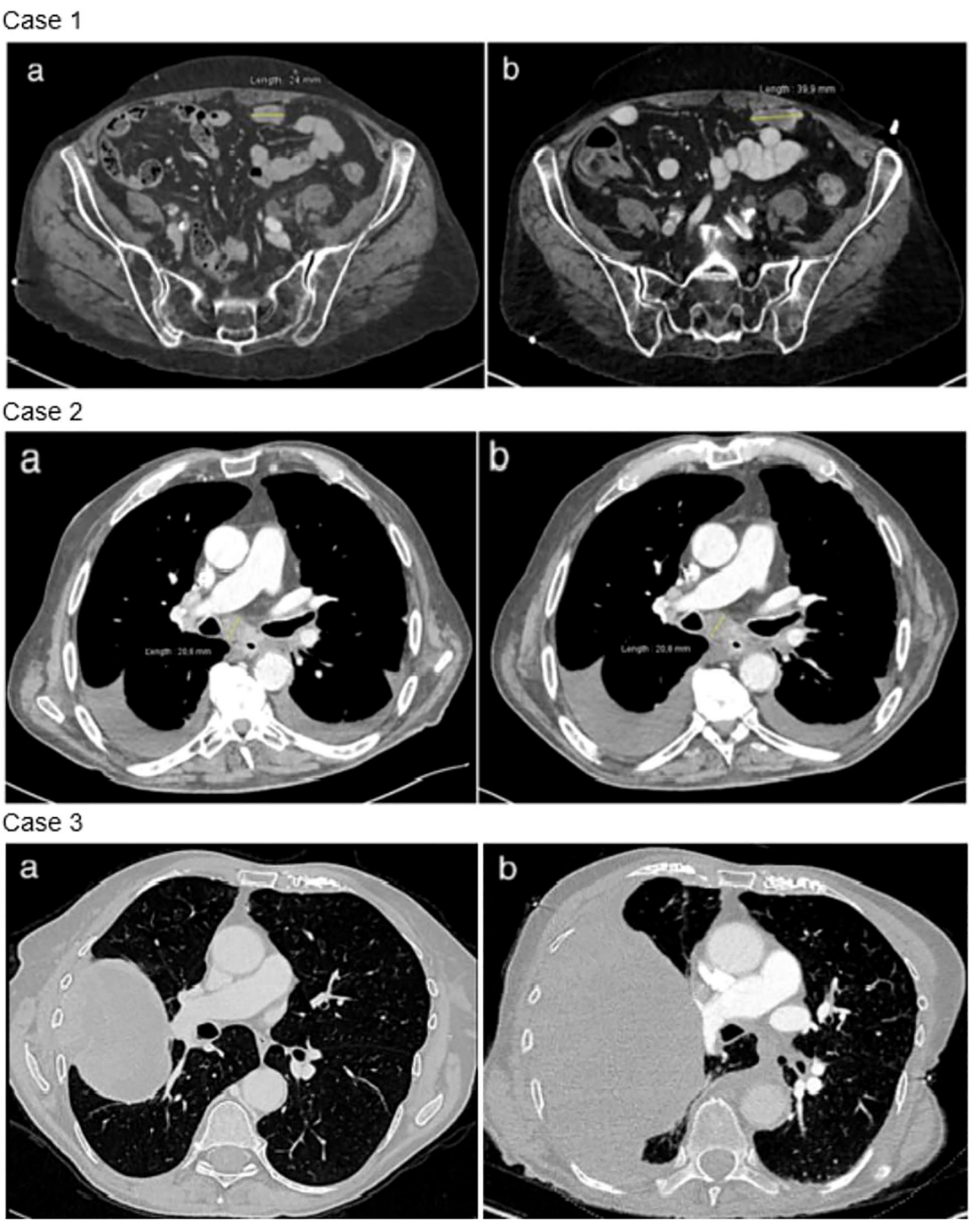
Radiological evolution under trametinib. Case 1: disease progression between baseline thoraco-abdominal computed tomography **(a)** and after 7 weeks of trametinib **(b)**. Example of a peritoneal metastasis. Case 2: stable disease between baseline thoraco-abdominal computed tomography **(a)** and after 6 weeks of trametinib **(b)**. Case 3: disease progression between baseline thoraco-abdominal computed tomography **(a)** and after 8 weeks of trametinib **(b)**.

Two pathogenic *NF1* mutations were identified (**Table 1**). Following TB proposition, the patient was started on trametinib in March 2023. At the onset of treatment, the patient had an ECOG of 2 and the Charlson score was 7 points. The treatment course was complicated by a grade 3 skin rash, requiring topical corticosteroid treatment and oral doxycycline. The first CT scan was performed at 7 weeks, showing progressive disease with a 70% increase in size of the lesions, per RECIST criteria (**Figure 3**). Treatment was discontinued, and the patient died rapidly.

### Case 2

The second case was an 80-year-old man, with a 65 pack-years smoking history. In January 2022, he was diagnosed with a metastatic adenocarcinoma PD-L1 negative. The initial treatment consisted in 4 cycles of carboplatin–pemetrexed–pembrolizumab administered. Due to her stable disease, maintenance with pemetrexed–pembrolizumab was started and then discontinued after one cycle due to grade 3 fatigue treatment-related reasons.

The patient was relatively well until November 2022, when he presented with pleural and pulmonary progression on his CT scan. Second-line treatment with paclitaxel and bevacizumab was started. The patient experienced poor clinical tolerance to chemotherapy with grade 3 fatigue, grade 4 thrombopenia, and grade 3 leucopenia. Further molecular analysis revealed a stop codon mutation in the *NF1* gene, as well as an increased copy number (4 copies) of the *NF1* gene. No alteration in RASA1 was found. Because of the allelic frequency, the mutated variant was likely located on the gained allele (**Table 1**). After discussion, and considering the poor patient tolerance to chemotherapy, a PS ECOG 2, and a Charlson score of 15 points, a treatment with trametinib was started in February 2023. The patient coped moderately with trametinib and complained of cough, and developed grade 2 creatinine elevation, grade 2 thrombocytopenia, and grade 1 anemia. All the side effects, except cough, were considered treatment related. None of these side effects required dose suspension or dose reduction.

After 6 weeks of trametinib, a CT scan showed stable disease per RECIST criteria (**Figure 3**). The clinical course was then complicated by an accidental fall and a hip fracture, followed by a heart failure with acute pulmonary edema. Trametinib treatment was immediately suspended, and the patient passed away 2 weeks later.

### Case 3

The third case was a 70-year-old woman, diagnosed with unresectable stage IIIC pulmonary adenocarcinoma of the right upper lobe, with a PDL1 expression of 10%. The patient underwent first-line chemo-immunotherapy, consisting of four cycles of carboplatin, pemetrexed, and pembrolizumab. After an initial partial response, the patient experienced progressive disease during his maintenance with pemetrexed–pembrolizumab.

Second-line treatment with paclitaxel and bevacizumab was administered for 3 cycles until January 2022. Molecular analysis revealed a mutation in a splice donor consensus sequence of the *NF1* gene with a gain of the mutated allele, based on the allele frequency and potentially resulting in partial NF1 inactivation (**Table 1**). No activating co-mutation of the RAS/MAPK pathway was identified, notably no RASA1 alteration. Subsequently, treatment with 2 mg of trametinib was initiated in February 2023. The patient had a ECOG 2 and a Charlson comorbidity index of 9 points. She experienced a grade 1 cutaneous rash and diarrhea. After 8 weeks of treatment, the CT scan showed thoracic and CNS progression (**Figure 3**). Trametinib was discontinued and, due to a rapid deterioration, the patient was started on best supportive care and died in April 2023.

### Case 4

The fourth case was a 62-year-old-man diagnosed with metastatic lung adenocarcinoma, with a PDL1 expression of 10%. The patient received first-line treatment with carboplatin, pemetrexed, and pembrolizumab with a partial response. After progression 1 year later, the patient had second-line treatment with carboplatin and gemcitabine for 3 months before experiencing further metastatic progression. Molecular analysis revealed a pathogenic mutation in the *NF1* gene with a loss of the other allele (**Table 1**). No co-mutation with *RASA1* was identified. The patient had an ECOG 2 and a Charlson comorbidity index of 7 points. Trametinib was initiated, but the patient experienced a very rapid deterioration of his general condition, resulting in death 1 week later. Clinical worsening occurred as a result of disease progression and was not attributed to trametinib toxicity.

## Discussion

*NF1* mutations have been described in both lung adenocarcinomas and squamous cell carcinomas, at rates of 8.3% and 12%, respectively (4), with a lower prevalence in Asian populations (9, 10). *NF1* is a large gene consisting of 60 exons (>280 kb, 60 exons), which can harbor a wide diversity of alterations—including nonsense, missense, frameshift, splice-site variants, indels, and large deletions—rendering comprehensive analysis technically challenging. This likely contributed to the relatively limited studies of NF1-mutant cancers compared with other oncogenic drivers. It was observed that most non-small cell lung cancer cases with *NF1* mutation do not exhibit co-mutations (16, 17). In the large genomic cohort analyzed by Bowman et al., *KRAS* mutations were the most frequent co-alteration (16.5%), followed by *EGFR* (6.8%) and *BRAF* (3.9%) alterations in white populations. These *KRAS* mutations were predominantly located in positions G12 and G13 (4). Similar patterns of co-mutations and smoking association were also described by Tlemsani et al. (18) In a previous observational study, most patients with *NF1* mutations were current or former smokers (88%). In the Asian population, a significantly higher number of co-mutations of *NF1* with known oncogenic mutations are described, predominantly involving *EGFR* mutations, of which we know that their prevalence in this population is higher (3). Whether *NF1* mutations carry a prognostic role in NSCLC remains unclear and appears to be context dependent.

Prognosis of patients with tumors carrying a *NF1*-inactivating mutation appear comparable with those with *KRAS mutations in codon 12 or 13* and less favorable than patients with mutated *EGFR* (4). Interestingly, in the Chinese population, an *NF1* mutation appears to be associated with a better prognosis in the case of the *EGFR* mutant/*TP53* wild type compared with those without the *NF1* mutation (19). In patients with mutated *EGFR*, the co-occurrence of a *TP53* mutation and an alteration in a tumor-suppressor gene such as *NF1* appears to have a worse prognosis (20). In a study by Liu et al., *NF1* mutations were associated with improved survival in patients receiving immunotherapy, but with poorer outcomes under targeted therapies for patients with *EGFR*-mutated or *ALK*-rearranged NSCLC (21). Other mutations co-occurring with *NF1* are worth noting like *RASA1*, a Ras-GTPase-activating protein (22), as *RASA1/NF1* co-mutation appears to be mutually exclusive with other driver mutations and to be more sensitive to MEK inhibition in preclinical models (15).

Literature on trametinib treatment for mutated *NF1* tumors is almost non-existent. The NCI-MATCH trial evaluated the efficacy of trametinib in patients with advanced neoplasia carrying an *NF1* mutation within one of its sub-protocols (7). Patients eligible for participation had advanced solid tumor, lymphoma, or multiple myeloma that had progressed after at least one line of systemic treatment. Three patients diagnosed with lung cancer were initially enrolled in the study; however, only one patient was evaluable for response, demonstrating an almost complete response with a median progression-free survival of 6.4 months. Notably, no co-mutations were identified in this case, notably no *KRAS* alteration (4). While both *NF1* loss and *KRAS* activating mutation lead to MAPK pathway activation and sensitivity to MEK inhibition *in vitro*, the use of MEK inhibitors has failed to demonstrate efficacy for patients with *KRAS-*mutated non-small cell lung cancer, likely due to the presence of several mechanisms of bypass of the MAPK pathway inhibition, such as increased AKT signaling in *KRAS*-altered tumors (23). In the National Lung Matrix trial, an umbrella study, the use of selumetinib, a MEK inhibitor, plus docetaxel in 14 patients with *NF1* loss showed an objective response rate of 31% with a median PFS of 5.3 months. Eligible patients received previous anticancer treatment or refused standard-of-care first line. Unfortunately, the isolated efficacy of selumetinib is not known in this study. The results may have been driven by the use of docetaxel (24). The trial NCT03232892 aimed to investigate the efficacy of trametinib in patients diagnosed with non-small cell lung cancer and bearing *NF1* mutations. Regrettably, the trial was prematurely terminated due to insufficient participant recruitment.

The four cases we described are therefore the largest cases series ever reported on the use of trametinib, a MEK inhibitor, in patients with metastatic lung adenocarcinomas harboring *NF1* alterations. All patients had altered performance status (ECOG score 1-2) at treatment initiation and received trametinib as a third-line treatment. Although some patients experienced severe adverse effects such as grade 3 skin rash and hematologic toxicities, these were managed effectively without requiring treatment discontinuation or dose reduction. This suggests that trametinib can be tolerated in this heavily pretreated population, provided careful monitoring is performed, including regular clinical assessments, blood counts, and liver and kidney function tests, with dose adjustments or temporary interruptions considered for grade ≥2 adverse events. However, careful monitoring remains essential. The treatment was moderately tolerated, but no effects required treatment discontinuation. Two out of three patients progressed on treatment according to RECIST criteria quite quickly at the first CT scan. The third patient achieved stable disease but sadly died after an accidental fall quite at the beginning of his treatment. The fourth patient died after only one week of trametinib treatment and could not be formally evaluated. In summary, one of three patients may have derived some radiological benefit from trametinib, although no deep response was seen.

A few factors may have contributed to the lack of response to trametinib in our cases. First and most importantly, two out of the four patients presented only one pathogenic mutation along with a gain of the *NF1* gene—probably the mutated allele, suggesting that the other wild-type allele might still be functional. In these cases, the monoallelic alteration of *NF1* might not be sufficient to activate the MAPK pathway on its own, therefore contributing to treatment resistance. Second, no concurrent alterations of the MAPK/ERK pathways were found in any patient. While certain mutations appear to confer resistance to MEK inhibitors in this scenario (e.g., *KRAS*), other alterations, such as loss of RASA1, have been shown to greatly enhance the sensitivity to MEK inhibition in NF1-deficient cell lines, highlighting maybe the importance of considering not only the *NF1* mutation but the entire genetic context. In patient 3, whether the *MAP3K13* amplification could have promoted a bypass survival mechanism through upregulation of the JNK signaling axis remains uncertain (25). In this perspective, it may be worthwhile in future research to explore combination therapies that address compensatory pathways or resistance mechanisms that limit the effectiveness of MEK inhibition in this setting. Finally, all patients were treated in the third-line setting and had altered performance status and short expected survival.

## Conclusions

We report four cases of metastatic lung adenocarcinoma with complete or partial NF1 inactivation treated with single-agent trametinib at the third line. None derived clinically significant benefit. Our observations question the use of trametinib in this population in particular in the presence of only one allele mutated, although the small number of patients, their heterogeneous molecular presentations, and the initiation of treatment at third-line therapy are important limitations. Comprehensive studies including more patients and a thorough analysis of all MAPK genes are necessary to advance our understanding of this subset of patients.

## Data Availability

The original contributions presented in the study are included in the article/supplementary material. Further inquiries can be directed to the corresponding author.
